# Cholera: Immunity and Prospects in Vaccine Development

**DOI:** 10.1093/infdis/jiy414

**Published:** 2018-09-01

**Authors:** Jason B Harris

**Affiliations:** 1 Division of Pediatric Global Health, Massachusetts General Hospital, Boston, Massachusetts; 2 Division of Infectious Diseases, Massachusetts General Hospital, Boston, Massachusetts; 3 Department of Pediatrics, Harvard Medical School, Boston, Massachusetts

**Keywords:** *Vibrio cholera*, cholera, oral cholera vaccines, immunity

## Abstract

*Vibrio cholerae* is a prototypical noninvasive mucosal pathogen, yet infection generates long-lasting protection against subsequent disease. Vibriocidal antibody responses are an imperfect but established correlate of protection against cholera following both infection and vaccination. However, vibriocidal antibody responses are likely a surrogate marker for longer-lasting functional immune responses that target the O-polysaccharide antigen at the mucosal surface. While the current bivalent inactivated oral whole cell vaccine is being increasingly used to prevent cholera in areas where the disease is a threat, the most significant limitation of this vaccine is it offers relatively limited direct protection in young children. Future strategies for cholera vaccination include the development of cholera conjugate vaccines and the further development of live attenuated vaccines. Ultimately, the goal of a multivalent vaccine for cholera and other childhood enteric infections that can be incorporated into a standard immunization schedule should be realized.

*Vibrio cholerae* is a comma-shaped, salt-tolerant, gram-negative bacteria that possesses a single sheathed flagellum and is highly motile. With the emergence of the seventh cholera pandemic, a dominant *V. cholerae* (El Tor biotype) lineage arose, which ultimately replaced the previously dominant sixth pandemic *V. cholerae* (classical biotype) strains [1]. While *V. cholerae* has been classified into >200 serogroups based on their O-polysaccharide antigen structure, only serogroups O1 and O139 have been associated with seventh pandemic *V. cholerae* [2]. *Vibrio cholerae* O139 emerged from a single horizontal transfer of the *rfb* locus into the seventh pandemic lineage and was a major cause of cholera in the 1990s and early 2000s but has since receded [3, 4]. The sixth pandemic *V. cholerae* lineage was also serogroup O1. The reasons why serogroup O1 strains have dominated as the cause of pandemic cholera are unknown, but these evolutionary constraints have tremendous relevance to immunity and cholera vaccines. *Vibrio cholerae* O1 is further subdivided into 2 serotypes, Inaba and Ogawa, which differ by a single methyl group in the terminal sugar of the O-antigen polysaccharide [5]. The additional methyl group is absent in the Inaba serotype due to inactivation of the *wbeT* methyltransferase [5].

*Vibrio cholerae* is human-restricted pathogen. It does not invade mucosal tissue, but it does penetrate the mucus lining of small bowel and anchors to the intestinal surface [6]. Unlike tissue invading gastrointestinal bacterial infections, *V. cholerae* does not cause clinically overt inflammation, but it does results in microscopic changes in the mucosal epithelium and in an innate immune response, including an influx of inflammatory cells and the production of innate effector molecules including lactoferrin, defensins, and oxidases [7]. Colonization requires the toxin co-regulated pilus, which provides a matrix for bacterial adherence and colony formation [8]. Expression of the toxin co-regulated pilus is coordinated with cholera toxin, and the pilus also serves as a receptor for the lysogenic bacteriophage CTXPhi, which allows toxin genes to be passed horizontally between *V. cholerae* [9]. Cholera toxin is an AB_5_ toxin. The B subunit (CtxB) pentamer binds to cell surface glycans, and the A subunit (CtxA) is cleaved and transported into the cell where it activates adenylate cyclase, causing the efflux of salt and water into the intestinal lumen [2]. In severe cholera, the outcome of this is vomiting, life-threatening watery diarrhea, and the excretion of trillions of organisms into the environment. The ingestion of 5 μg of cholera toxin is sufficient to cause the symptoms of cholera [10].

## Immunity Following Infection

*Vibrio cholerae* induces long-lasting immunity in most people who recover from infection. This has been observed in US volunteers infected with wild-type *V. cholerae* O1 then challenged later with a second dose of bacteria. These challenge studies demonstrate that a single episode of experimental *V. cholerae* infection resulted in 100% protection against reinfection with either the homologous or heterologous serotype for at least 3 years—the longest interval tested [11].

Decades of surveillance in Matlab, Bangladesh, also demonstrate that *V. cholerae* infection results in long-lasting immunity [12, 13]. An evaluation of hospitalizations in Matlab between 1968 and 1977 identified only 3 repeat hospitalizations from cholera, all in young children; suggesting that infection resulted in an approximately 90% protection against subsequent disease [13]. A second retrospective study in Matlab, between 1991 and 2000, found that infection conferred 65% protection over the 3 years following (95% confidence interval, 37%–81%) relative to age-matched controls. However, while *V. cholerae* O1 Inaba infection conferred complete protection against either serotype, there was no evidence of cross-protection against Inaba if the original infection was caused by the Ogawa serotype [12]. This suggests that serotype heterotypic immunity may be more complex than previous human challenge studies suggest.

## Antigenic Repertoire of *V. cholerae*

Research on adaptive immunity to cholera has focused primarily on antibodies, as antibody responses are thought to mediate protection at the mucosal surface. Antibody responses are directed at the O1-specific polysaccharide component of the lipopolysaccharide and cholera toxin [14]. When early B-cell responses following cholera were evaluated at the single-cell/monoclonal antibody level in adults living in an endemic area, >75% of clonally expanded plasmablasts produced antibodies that targeted the O1-specific polysaccharide or cholera toxin antigens [15]. The O1-specific polysaccharide antibodies had varying degrees of cross-reactivity against the Inaba and Ogawa serotypes, suggesting a mechanism for the varying degree of cross-serotype immunity following infection [15].

In this same study, monoclonal antibodies to cholera toxin targeted both CtxA and CtxB, and were almost all cross-reactive with the enterotoxigenic *Escherichia coli* heat-labile toxin; effectively blocking the function of both cholera toxin and heat-labile toxin in vitro [15]. Utilizing an antigen microarray, a sialidase, NanH, was identified as a third dominant target of the humoral immune response to cholera [15, 16], though other proteins also contributed to the antigenic repertoire of *V. cholerae* to a lesser extent [15, 16]. Overall, these findings suggest that a small number of key antigens dominate the immune response to cholera despite the complex structure and composition of the organism.

## MECHANISMS AND CORRELATES OF PROTECTION AGAINST CHOLERA

### Antitoxin Responses

Surprisingly, there is little evidence that antibody responses to cholera toxin contribute to long-term protective immunity, but they do appear to contribute to short-term protection. Although antitoxin antibodies block toxin function in vitro and are protective in animal models of cholera, higher levels of circulating CtxB-specific immunoglobulin G (IgG) antibodies and CtxB-specific IgG memory B cells are not associated with protection against cholera [17–19]. Specifically, there is no association between CtxB responses and protection in human challenge studies following vaccination [19], and no association between CtxB IgG or memory B-cell responses and the risk of infection in household contacts of patients with cholera [17, 18].

However, higher levels of circulating CTB immunoglobulin A (IgA) antibodies are indeed correlated with protection against cholera, though these responses are short-lived [17]. This is consistent with studies that demonstrate that oral cholera toxoid vaccine provides only limited, short-term protection against severe cholera [20]. This is also consistent with data from vaccine trials that demonstrate that while an oral inactivated whole cell vaccine containing CtxB provides more protection than a whole cell-only vaccine in the first year following vaccination, no additional benefit is observed from the addition of CtxB beyond 1 year postvaccination [21].

### Vibriocidal and Anti–O Polysaccharide Antibody Responses

In contrast to antitoxin responses, antibacterial responses are clearly important for long-term protective immunity against cholera. Currently, the best correlate of protection against cholera is the vibriocidal antibody titer, which measures the minimum concentration of serum required for antibody-dependent complement-mediated bacterial killing [17, 19, 22]. Several studies demonstrate that the vibriocidal response primarily targets the O antigen, though other antigens may contribute to a lesser extent [15, 23, 24].

However, vibriocidal antibodies are both an unlikely and imperfect marker of protection against cholera. While antibody-dependent complement-mediated bacterial killing is an important defense against systemic infections, it is not thought to be important at the intestinal surface owing to low levels of complement at this site [25]. Instead, the inhibition of motility and trapping of bacterial pathogens in the mucus layer by dimeric IgA are considered likely effector functions of antibodies to prevent colonization by *V. cholerae* [26, 27].

This lack of a direct mechanistic connection between circulating vibriocidal antibodies and protective immunity probably explains its limitations as a correlate of protection [25]. While vibriocidal antibody titers increase with age in cholera-endemic areas, and higher vibriocidal titers are associated with a decreased risk of infection, there is no threshold titer at which protection is universally achieved [25]. Moreover, in human challenge studies, circulating vibriocidal antibody titers generally decrease to low or baseline levels before protective immunity to cholera wanes [19]. Despite these limitations, the vibriocidal antibody response appears to be a useful proxy marker for a longer-lasting functional mucosal immune response that targets the O antigen.

### Persistence of Immune Memory After *V. cholerae* Infection

There are at least 2 possible explanations for the persistence of protective immunity against cholera even in individuals who have low levels of circulating antibody. First, immunity may be maintained by long-lived secretory IgA (sIgA)–producing plasma cells at the mucosal surface. However, evidence from intestinal biopsies from patients recovering from cholera suggests that O1 antigen–specific IgA secretion falls sharply within 6 months after infection [28].

Alternatively, protective immunity may be maintained in the absence of high levels of mucosal sIgA by memory B (B_M_) cells. B_M_ cells are long-lived cells that do not produce antibody, but can rapidly expand and differentiate into plasmablasts upon antigen exposure. Against *V. cholerae* infection, B_M_ cells could provide immunity by generating a sufficiently rapid response. In support of this, household contacts with circulating O antigen–specific B_M_ cells were much less likely to be infected with *V. cholerae*, even if they had no evidence of circulating vibriocidal antibodies at the time of exposure [18]. Similarly, O antigen–specific B_M_ responses following vaccination were associated with protection against infection in US participants in human challenge study described above [19].

## CHOLERA VACCINES AND IMMUNITY: PAST, PRESENT, AND FUTURE

Robert Koch reported his discovery and isolation of the cholera bacillus in 1884, and within a year the first cholera vaccine, an injection of cultured unattenuated *V. cholerae*, was tested during an outbreak in Valencia, Spain. The vaccine had protective efficacy of 80% [29]. Subsequently, several inactivated whole cell vaccines were developed, which resulted in short-term protection but produced significant local reaction at the site of injection and high rates of fever and malaise among vaccinees [30].

### Current Cholera Vaccines

The injectable whole cell cholera inactivated vaccines have since been replaced by the current generation of oral cholera vaccines. This includes both live-attenuated and inactivated oral whole cell vaccines, which were first developed the 1980s. In contrast with whole cell injectable vaccines, these oral cholera vaccines are safe and well tolerated [2].

### Whole Cell–Recombinant B Subunit Vaccine

The first widely used oral vaccine was the whole cell–recombinant B subunit (WC-rBS) vaccine (CtxB). WC-rBS is currently manufactured as Dukoral by Valneva, primarily as a vaccine for travelers. The WC-rBS vaccine is derived from a mix of heat- and formalin-inactivated whole cells, with a complex composition that includes inactivated sixth and seventh pandemic strain *V. cholerae* O1 of both serotypes and a milligram of recombinant CtxB.

The most impactful field trial of the WC-rBS vaccine was conducted in 1985 in Matlab, Bangladesh, in children aged 2–15 years and women aged ≥15 years [21, 31–33]. The trial used a precursor to the current vaccine, which contained purified rather than recombinant CtxB. The trial demonstrated a cumulative 50% protective efficacy over 3 years, but limited, short-term protection in children <5 years old, and no protection in adults beyond 3 years [32].

A recent study of the WC-rBS vaccine demonstrated limited B_M_ cell responses and limited O-antigen responses in young children [34]. Vaccination with WC-rBS also results in CD4 T-cell responses skewed toward a Treg (tolerance inducing) response rather than the T_H_1 and T_H_17 response associated with natural infection [35]. This could be due to the immunomodulatory properties of CtxB [35]. In support of this, in the 1985 Matlab trial, which included a comparison of the whole cell–only and whole cell with CtxB arms, the whole cell–only version of the vaccine resulted in longer-lasting immunity, although the CtxB component conferred better short-term immunity (especially in young children) [21, 33, 36].

### Bivalent Inactivated Whole Cell Vaccine

A more recently developed bivalent inactivated whole cell vaccine (bivWC) is produced without CtxB. The bivWC vaccine is less expensive and has been increasingly used to prevent or respond to cholera epidemics and in cholera-endemic areas. Currently, the bivWC vaccine is manufactured as Shanchol by Shantha Biotechnics and Euvichol by EuBiologics. While bivWC includes serogroups O1 and O139, the latter has not been a cause of pandemic cholera in more than a decade.

While there have been no direct head-to-head trials comparing the bivWC and WC-rBS vaccine, bivWC appears to generate higher fold increases in vibriocidal antibody titer following vaccination [33, 37, 38] and more robust O antigen–specific B_M_ cell responses (Falkard et al., unpublished data) and, perhaps because of this, appears to generate longer-lasting protection than the WC-rBS vaccine in adults [39]. However, like the WC-rBS vaccine, the bivWC vaccine provides more limited protection in young children [40]. Interestingly, immunogenicity studies of bivWC vaccine demonstrate that vibriocidal responses in children aged >2 years are similar to those in older children and adults, but vibriocidal responses to vaccination were limited in children <2 years of age [41]. More extensive studies would be needed to assess the extent of the immunogenicity and protection associated with the bivWC vaccine across the age spectrum of young children with sufficient granularity.

### CVD-103-HgR (Oral Cholera Vaccine)

The attenuated oral cholera vaccine, CVD-103-HgR, was also developed in the 1980s, and previously licensed as Orochol in the 1990s. A remanufactured version of this vaccine is now available in the United States as Vaxchora (PaxVax). This vaccine is recommended by the Advisory Committee for Immunization Practices for US travelers to areas with active cholera transmission [42]. The CVD-103-HgR vaccine was derived from a sixth pandemic O1 Inaba strain by deletion of the CtxA subunit and addition of a mercury resistance marker [43]. The efficacy of CVD-103-HgR has been evaluated primarily through human challenge studies in US participants [22, 43, 44].

In areas where cholera is endemic, the vibriocidal responses to CVD-103-HgR vaccine are lower, but can be improved by reformulating the vaccine with a higher number of organisms [45]. While this reformulated CVD-103-HgR vaccine had an effectiveness of 80% (similar to the protection achieved by inactivated whole cell vaccines) during a cholera epidemic in Micronesia [46], the vaccine was not found to be effective in a single randomized trial in Indonesia [47]. While in retrospect high rates of vaccine coverage could have masked the efficacy of this vaccine due to herd immunity, additional evaluation is needed in areas where cholera is endemic [48].

### Approaches to Future Cholera Vaccines

Cholera vaccination is an essential part of cholera control programs, and vaccination programs prevent cholera and save lives in areas where cholera is a threat. However, like other vaccines, it is possible that cholera vaccines can either be improved or new vaccines can be developed that are tailored to specific groups of people (eg, a specific vaccine for young children vs older children and adults). The importance and immunogenicity of cholera vaccines in individuals with HIV infection is also discussed separately in Table 1.

The most important and pressing limitation is that none of the currently available oral cholera vaccines are associated with established long-term immunity in young children [40]. This is critical given that children are most vulnerable to cholera where the disease is endemic [17, 49]. Not surprisingly, the limited effectiveness of the inactivated oral cholera vaccines in young children is associated with impaired responses to the O-polysaccharide antigen [41, 50].

### Conjugate Vaccines

One strategy to overcome the poor O-polysaccharide antigen response in young children is through the development of an O1-polysaccharide antigen conjugate vaccine. This approach has been applied to develop effective vaccines for young children for several invasive encapsulated bacterial infections including pneumococcal, *Haemophilus influenzae*, and meningococcal infections, as well as the enteroinvasive pathogen *Salmonella enterica* serovar Typhi [51, 52]. However, while these conjugate vaccines have demonstrated efficacy against invasive bacterial infections, the effectiveness of this approach for a noninvasive mucosal infection has not yet been established in humans.

The first O1 conjugate vaccines developed were bound to cholera toxin and stimulated longer-lasting vibriocidal antibody responses (compared to a nonconjugate version) in a phase 1 clinical trial [53]. More recently *V. cholerae* O1 conjugate vaccines have been produced using squaric acid chemistry, which has advantages in terms of antigen display and manufacturing costs [54–56]. Although an O1 Inaba conjugate vaccine administered intramuscularly to mice failed to elicit a lamina propria IgA response [56] other strategies could be used to aid in targeting these otherwise robust immune responses toward the mucosal barrier. For example, a sequential prime-boost strategy (of oral vaccination followed by intramuscular conjugate vaccine) or the use of mucosal adjuvants (such as mutant heat-labile toxin) could be explored to develop a conjugate vaccine against a noninvasive pathogen [57].

### Live Attenuated Vaccines

The further development of live attenuated vaccines has the potential advantages of better mimicking the innate immune responses associated with infection, as well as generating responses to in vivo expressed antigens which may contribute to long-term protection [58]. Other approaches to improve on live attenuated cholera vaccines have focused on using genetic modifications in more recent pandemic strains to limit risk of the horizontal exchange of virulence genes among vaccine strains [59]. Interestingly, a new attenuated vaccine strain, engineered from the recent seventh pandemic *V. cholerae* El Tor isolate, was shown to function effectively as probiotic in the short term by blocking colonization by virulent *V.* cholerae in an infant rabbit model of cholera [60]. If this also occurs in humans, and the attenuated strain is sufficiently immunogenic, then this probiotic effect many represent a previously unrecognized potential benefit from a live attenuated cholera vaccine, particularly in the setting of an active outbreak of cholera [61].

### Other Approaches

Other approaches to improve cholera vaccines may facilitate vaccination by lowering the cost of vaccine production or by producing a vaccine that can be more effectively integrated into a standard childhood immunization schedule in areas where cholera is a threat. For example, the recent development of *V. cholerae* O1, which stably expresses both the Inaba and Ogawa serotype antigens, provides a potential platform to produce a lower-cost inactivated vaccine, relative to the more complex bivWC vaccine formulation [62]. Vaccination with *V. cholerae* outer membrane vesicles has also been proposed as an alternative strategy [63, 64].

Another strategy to improve on cholera vaccines might include the development of a multivalent vaccine to protect broadly against childhood enteric disease. As the mortality from childhood diarrhea continues to decline, we are only beginning to appreciate the tremendous morbidity and developmental impact of a small number of bacterial, viral, and parasitic pathogens that contribute to the burden of childhood enteric infection [65, 66]. Ultimately, the goal of producing a multivalent conjugate vaccine for childhood enteric infection, or the engineering of an attenuated heterologous antigen–expressing vaccine, must be within reach given sufficient resources. In the vaccine–pathogen arms race, it is unlikely that the human immune system will be the limiting factor in the development of the next generation of vaccines to cholera and other intestinal pathogens.

**Table 1. Human Immunodeficiency Virus, Cholera, and Vaccines**

Studies of the prevalence of human immunodeficiency virus (HIV) infection among patients presenting with cholera during the 2005 epidemic in Mozambique and the 2010 epidemic in Haiti suggest that individuals living with HIV infection have an increased risk of severe cholera relative to the rest of the population [67, 68]. Although no studies have directly addressed the efficacy of cholera vaccination in individuals with HIV infection, oral cholera vaccines are generally safe and immunogenic, although vibriocidal responses following bivalent inactivated whole cell vaccination were found to diminish among individuals with CD4^+^ T-cell counts of <350 cells/μL [69]. In a study of CVD-103-HgR infection in adults in Mali, seroconversion rates were slightly lower in adults with HIV infection, but there was no difference in the occurrence of adverse events in the adults with and without HIV infection [70].

## Notes

***Disclaimer.*** The funding source had no role in the writing or content of this manuscript.

***Supplement sponsorship.*** This work is part of a supplement sponsored by the Bill and Melinda Gates Foundation.

***Financial support.*** J. B. H. received funding from the National Institutes of Health (grant numbers AI103055 and AI099243).

***Potential conflicts of interest.*** Author certifies no reported conflicts of interest. The author has submitted the ICMJE Form for Disclosure of Potential Conflicts of Interest. Conflicts that the editors consider relevant to the content of the manuscript have been disclosed.
